# Imaging of Lymph Node Micrometastases Using an Oncolytic Herpes Virus and [^18^F]FEAU PET

**DOI:** 10.1371/journal.pone.0004789

**Published:** 2009-03-10

**Authors:** Peter Brader, Kaitlyn Kelly, Sheng Gang, Jatin P. Shah, Richard J. Wong, Hedvig Hricak, Ronald G. Blasberg, Yuman Fong, Ziv Gil

**Affiliations:** 1 Department of Radiology, Memorial Sloan-Kettering Cancer Center, New York, New York, United States of America; 2 Department of Surgery, Memorial Sloan-Kettering Cancer Center, New York, New York, United States of America; 3 Department of Neurology, Memorial Sloan-Kettering Cancer Center, New York, New York, United States of America; 4 The Laboratory for Applied Cancer Research, Tel Aviv Sourasky Medical Center, Tel Aviv University, Tel Aviv, Israel; 5 Department of Radiology, Medical University Graz, Graz, Austria; Karolinska Institutet, Sweden

## Abstract

**Background:**

In patients with melanoma, knowledge of regional lymph node status provides important information on outlook. Since lymph node status can influence treatment, surgery for sentinel lymph node (SLN) biopsy became a standard staging procedure for these patients. Current imaging modalities have a limited sensitivity for detection of micrometastases in lymph nodes and, therefore, there is a need for a better technique that can accurately identify occult SLN metastases.

**Methodology/Principal Findings:**

B16-F10 murine melanoma cells were infected with replication-competent herpes simplex virus (HSV) NV1023. The presence of tumor-targeting and reporter-expressing virus was assessed by [^18^F]-2′-fluoro-2′-deoxy-1-β-D-β-arabinofuranosyl-5-ethyluracil ([^18^F]FEAU) positron emission tomography (PET) and confirmed by histochemical assays. An animal foot pad model of melanoma lymph node metastasis was established. Mice received intratumoral injections of NV1023, and 48 hours later were imaged after *i.v.* injection of [^18^F]FEAU. NV1023 successfully infected and provided high levels of *lacZ* transgene expression in melanoma cells. Intratumoral injection of NV1023 resulted in viral trafficking to melanoma cells that had metastasized to popliteal and inguinal lymph nodes. Presence of virus-infected tumor cells was successfully imaged with [^18^F]FEAU-PET, that identified 8 out of 8 tumor-positive nodes. There was no overlap between radioactivity levels (lymph node to surrounding tissue ratio) of tumor-positive and tumor-negative lymph nodes.

**Conclusion/Significance:**

A new approach for imaging SLN metastases using NV1023 and [^18^F]FEAU-PET was successful in a murine model. Similar studies could be translated to the clinic and improve the staging and management of patients with melanoma.

## Introduction

Solid tumors including malignant melanoma, breast, head and neck, gastrointestinal and genitourinary carcinomas are prevalent cancers worldwide and are a primary cause of mortality in the Western world [1]. These tumors frequently metastasize to regional lymph nodes, and lymph node invasion by cancer cells is one of the most significant factors in predicting prognosis [2]. Even in the absence of clinical or radiologic evidence of lymph node metastases, the risk for positive regional lymph nodes is frequently high enough to necessitate lymph node dissection [3]. Surgery for removal of regional lymph nodes is indicated not only for ablation of cancer, but also for evaluation of the need for radiation therapy or chemotherapy. The patterns of cancer spread into lymph nodes are predictable, based on the anatomic location of the primary tumor. Therefore, a selective surgical procedure is commonly directed to the basins at risk for lymphatic spread [4]. The morbidity associated with and costs of lymph node dissections are substantial. Nevertheless, conventional imaging techniques are unsatisfactory for the evaluation and localization of regional lymph node metastases due to their limited ability to identify subcentimeter micrometastases. Thus, there is a need for an effective noninvasive modality that can accurately identify occult lymph node metastases in these patients [5].

**Figure 1:**
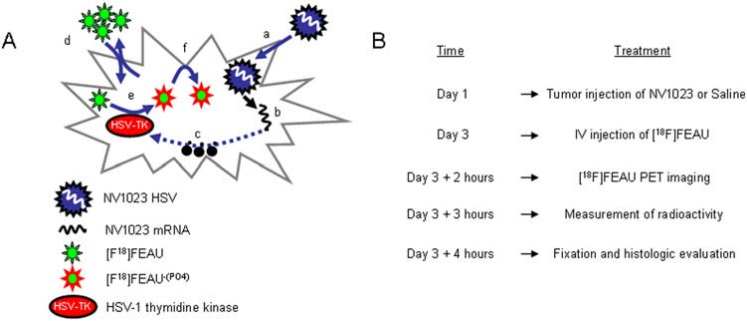
Schematic demonstration **of (A) virus infection, replication, transgene expression, and [18F]FEAU PET imaging and (B) *in vivo* experimental setup.**

Noninvasive reporter gene imaging has been successfully applied to monitoring gene therapy, including gene delivery and expression mediated by retroviral [6], adenoviral [7], and oncolytic herpes viral vectors [8], [9], [10]. The concept of using herpes oncolytic viruses and positron emission tomography (PET) imaging is based on the ability of these vectors to selectively infect cancer cells and not stromal tissue [11], [12]. After entering the cell, viruses readily express their early genes, among them the herpes simplex virus (HSV) type 1 (HSV-1)*–thymidine kinase* (*tk*) gene product. As opposed to the mammalian *tk*-1, the HSV1-*tk* gene product has the ability to catalyze the phosphorylation of [^18^F]-2′-fluoro-2′-deoxy-1-β-D-β-arabinofuranosyl-5-ethyluracil ([^18^F]FEAU). Phosphorylated [^18^F]FEAU, is trapped inside HSV1-*tk* expressing cancer cells; it accumulates in proportion to the level of HSV1-*tk* expression and can be imaged using PET (**Figure 1A**).

The purpose of this study was to develop a noninvasive method for the detection of micrometastases in regional lymph nodes using a replication-competent oncolytic virus carrying the *HSV-1-tk* gene. We show, for the first time, that oncolytic viruses introduced into the lymphatic system via single intratumoral injection can be used efficiently to detect melanoma micrometastases in regional lymph nodes using [^18^F]FEAU PET imaging.

## Materials and Methods

### Cell lines

The syngeneic malignant melanoma cell line B16-F10 was used for the *in vivo* and *in vitro* experiments. Cells were grown in Dulbecco's modified Eagle's medium (DMEM) supplemented with high glucose (HG), 10% fetal calf serum (FCS), 1.5 mmol/L sodium bicarbonate, 100 µg/L penicillin, and streptomycin. Cells were maintained in 5% CO_2_ in a 37°C humidified incubator.

### Viruses

NV1023 is an attenuated, replication-competent oncolytic HSV whose construction was previously described [11]. In brief, this virus was derived from R7020, an HSV-1 (F′ strain) vector originally designed as an HSV vaccine. The virus carries a 5.2-kB fragment of HSV-2 DNA (containing HSV-2 genes *US2-2* through *US2-5*) inserted in the UL/S junction. Attenuation was achieved by a 15-kB deletion in the inverted repeat region that extends from the 3′ end of *UL55* to the promoter for *ICP4*, deleting *UL56* and 1 copy of the diploid genes *ICP0* and *ICP4* and the neurovirulence gene γ*_1_34.5*. The *Escherichia coli* β-galactosidase gene (*lacZ*) was inserted at the US10-12 locus as an infection marker.

### β-galactosidase assays

Cancer cells were plated at 1×10^4^ cells per well in 96-well plates in 100 µL medium. After incubation for 6 hours, NV1023 (500 µL) was added to each well at a multiplicity of infection (MOI, or the ratio of viral particles per tumor cell) of 0.1. Expression of the *lacZ* gene was measured at 24-hour intervals with an enhanced β-galactosidase assay kit (Gene Therapy Systems, San Diego, CA) using a spectrophotometer at 570 nm. All samples were measured in triplicate.

### *In vivo* foot pad model

Animal studies were performed in compliance with all applicable policies, procedures and regulatory requirements of the Institutional Animal Care and Use Committee (IACUC), the Research Animal Resource Center (RARC) of MSKCC and the National Institutes of Health (NIH) “Guide for the Care and Use of Laboratory Animals”. All animal procedures were performed by inhalation of 2% isoflurane. After the studies all animals were sacrificed by CO_2_ inhalation.

A foot pad syngeneic melanoma model was established as described previously by Harrell et al [13]. Briefly, 30 6-week-old C57BL/6J-Tyr^C-2J^/J albino mice (Jackson Laboratories, Bar Harbor, Maine) were anesthetized with inhalational isoflurane for all procedures. The right hind limb foot pad was sterilized with alcohol, and then slowly injected with 50 µL of cell suspension at a concentration of 2×10^5^ cells/µL over a 2-minute period. The mice were then awakened and their foot pad monitored for tumor size and signs of pain or ulceration twice a week. Three to 4 weeks after tumor injection we could monitor the presence of isolated black-colored sub centimeter lymph node at the popliteal area, i.e., the first echelon lymphatic region of the foot pad. In some animals there was also evidence for positive lymph nodes in the inguinal area, the secondary echelon of the foot pad. Only animals with primary tumors <5 mm that were confined to the foot pad area (50% of the animals) were used in the PET imaging study. NV1023 (1×10^7^ viral plaque-forming units [PFU]) was injected to the foot pads bilaterally (both to the tumor and the contralateral foot pad). Five of the animals with tumors were injected with saline before [^18^F]FEAU/PET imaging and served as a negative control. Another 5 animals without tumors were injected with NV1023 and the morphology of their lymph nodes was monitored.

Histologic examination confirmed the presence of metastatic melanoma cells in cases of palpable/black-colored nodes, which were generally 0.8 mm in dimension at the time of diagnosis. Metastatic melanoma cells were deposited in the subcapsular sinus of the lymph node and infiltrated the nodal parenchyma.

### [^18^F]FEAU PET

The concept of [^18^F]FEAU PET imaging with NV1023 is based on the ability of the HSV-1-*tk* reporter gene, to catalyze the phosphorylation of the thymidine analog [^18^F]FEAU. **Figure 1** depicts the mechanism by which the HSV-1-*tk* transgene serves as a molecular marker for the detection of melanoma metastases.

[^18^F]FEAU tracer was synthesized by coupling the radiolabeled fluoro sugar with the silylated pyrimidine derivative following a procedure previously reported by Serganova [14]. The specific activity of the product was ∼37 GBq/µmol (∼1 Ci/µmol); radiochemical purity was >95% following purification by high-performance liquid chromatography (HPLC).

Following virus injection, animals were intravenously injected with ∼250 µCi (9.25 MBq) of [^18^F]FEAU tracer 2 hours before PET imaging was performed. Mice were sacrificed with CO_2_, degutted, and immediately imaged with a dedicated small-animal PET scanner (Focus 120 micro-PET, Concorde Microsystems, Knoxville, TN). The foot pad of the animals was amputated prior to imaging to avoid high background levels of radiotracer uptake in the tumor. In all animals the tumor was localized to the foot pad and did not infiltrate below the wrist. Images were acquired using a transaxial field of view of 10 cm and an axial field of view of 7.8 cm. An energy window of 350–750 keV and a coincidence timing window of 6 nanoseconds were used. The resulting list-mode data were sorted into 2-dimensional histograms by Fourier rebinning, and transverse images were reconstructed by filtered backprojection into a 128×128×63 (0.72×0.72×1.3 mm) matrix. Image analysis was performed using the ASIPro (Siemens Preclinical Solutions, Knoxville, TN). After imaging, the popliteal and inguinal lymph nodes were excised bilaterally, weighed, and measured for radioactivity (%ID/g) using a gamma counter (Packard, United Technologies, Downers Grove, IL).

### Histochemistry

After the final image, the animals were sacrificed and the lymph nodes harvested and frozen in Tissue-Tek Optimal Cutting Temperature (OCT) compound (Sakura Finetek USA, Inc., Torrance, CA). Tissues were cut into 5-µm-thick sections and mounted on glass slides. Cryosections were fixed and lymph nodes were stained with hematoxylin and eosin (H&E) or 5-bromo-4-chloro-3-indolyl-b-D-galactopyranoside (X-gal) for assessment of β-galactosidase expression. Cells were stained for 4 hours with X-gal (1 mg/mL) in an iron solution of 5 mM K_4_Fe(CN)_6_, 5 mM K_3_Fe(CN)_6_, and 2 mM MgCl_2_, as previously described. Counterstaining of background cells with nuclear fast red was performed. Virally infected cells expressing β-galactosidase were identified histologically as blue-staining cells.

Criteria for lymph node status were based on microscopic evaluation: *a*) lymph nodes with melanoma metastases that were readily identified by the presence of black melanin pigment were considered positive nodes; and *b*) lymph nodes that were devoid of cancer cells were considered negative nodes.

### Viral Proliferation

For the viral proliferation studies, 2×10^4^ cells were seeded per well in 12-well plates in 1 mL medium. After incubation for 6 hours, NV1023 (100 µL) was added to each well at MOIs of 0.01, 0.1, and 1. Supernatant containing released virus was collected at day 2 and frozen. Vero cells were grown to confluence on 6-well plates. Supernatant samples were thawed, and samples were diluted 10-fold serially. Dilutions were then incubated on confluent Vero cells for 4 hours. Wells were washed with medium and covered with 1% agarose in medium. After 48 hours, 2 mL of neutral red solution (2% by volume) was added, and viral plaques were counted 24 hours later. Samples were done in triplicate.

### Immunoblotting

For expression of β-galactosidase and HSV1TK, cells were grown in 6 well plates. Forty-eight hours after infection with NV1023 at MOIs of 0.01, 0.1 and 1, cells were released without enzymatic digest at 4°C. Cell pellets were sonicated for 10 seconds and clarified by centrifugation. Total protein (50 µg) underwent electrophoresis in 7.5% Tris-HCl gels (Bio-Rad, Hercules, CA) and was transferred to polyvinylidene difluoride membranes, blocked and exposed to primary antibody followed by a secondary antibody conjugated to horseradish peroxidase. Bands were developed using an ECL Plus detection system (Amersham, Piscataway, NJ). Density was quantified using a computer-controlled CCD camera (AlphaImager Imaging Systems, Alpha Innotech, San Legndra, CA). The following antibodies were used: β-actin, HSV1-TK and β-galactosidase (Santa Cruz Biotech, Santa Cruz, CA)

### Statistical Analysis

Student *t* tests or analyses between groups were used for statistical analysis as appropriate. Differences were considered significant at *P*<0.05. All data is represented as mean±SD, unless otherwise indicated. All experiments were repeated in triplicate. Data from representative experiments are shown.

## Results

### Time course of NV1023 infection

**Figure 2:**
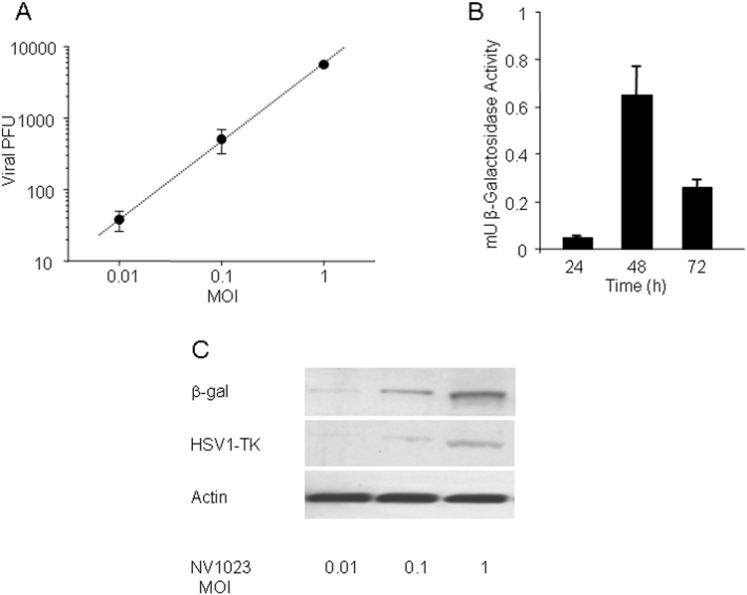
*In vitro *β-galactosidase and HSV-1TK transgene expression over time. (A) Viral amplification assay. Cells were infected with NV1023 at MOIs of 0.01–1 and viral titers measured at 48 hours. NV1023 demonstrated highly efficient replication in B16-F10 melanoma cells within 48 hours after infection. (B) β-galactosidase activity in B16-F10 melanoma cells at 24, 48, and 72 hours after infection with NV1023. (C) Western blotting analysis performed 48 hours after viral infection at MOIs of 0.01–1 revealed that expression of β-galactosidase was correlated with HSV1-tk expression in melanoma cells.

We first evaluated the ability of NV1023, a replication-competent HSV, to infect malignant melanoma cells *in vitro* using a viral amplification assay. Cells were infected with NV1023 at MOIs of 0.01–1 and viral titers measured at 48 hours. NV1023 demonstrated highly efficient replication in B16-F10 melanoma cells within 48 hours after infection. As shown in **Figure 2A**, melanoma cells strongly supported viral proliferation, with an approximately 100-fold increase in viral titer from MOI of 0.01 to 1.

The *Escherichia coli* β-galactosidase transgene (*lacZ*), inserted at the US10-12 locus of the NV1023 virus, was used as an infection marker. The ability of NV1023 to infect B16-F10 melanoma cells was studied *in vitro* during a 3-day period. The expression of the *lacZ* gene increased more than 13-fold during the first 48 hours after inoculation and decreased 72 hours after infection (**Figure 2B**). Western blotting analysis performed 48 hours after viral infection at MOIs of 0.01–1 revealed that expression of β-galactosidase was correlated with HSV1-tk expression in melanoma cells (**Figure 2C**). Based on this data, we postulated that the optimal time for imaging of virus infection would be approximately 48 hours after inoculation.

### Identification of lymph nodes with melanoma metastases and expression of viral proteins

**Figure 3:**
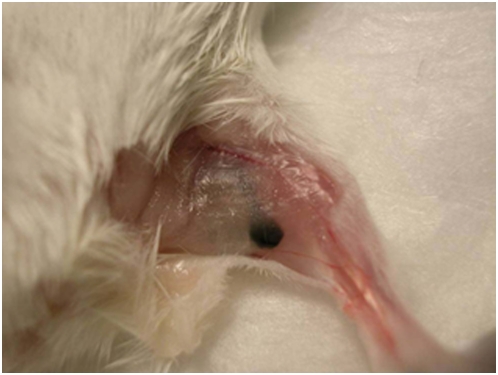
In situ photograph of lymph node metastasis from melanoma. B16-F10 melanoma cells were injected into the right footpad of C57BL/6J-TyrC-2J/J mice. Twenty days after cancer cell injection, melanoma metastases were detected in the right popliteal area.

Malignant melanoma tumors were established in the right foot pad of 30 C57BL/6J-Tyr^C-2J^/J albino mice by direct injection of 2×10^5^ melanoma cells (B16-F10). Twenty days after cancer cell injection, approximately 50% of the animals had evidence of subcentimeter black-colored lymph nodes in their popliteal area (**Figure 3**).

**Figure 4:**
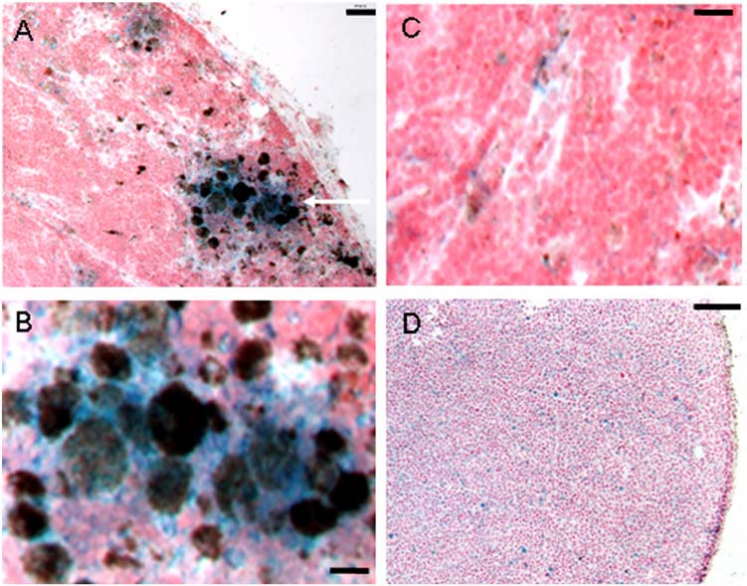
Histologic analysis of lymph nodes after imaging showing metastatic and normal lymph nodes from mice injected with NV1023. (A) *LacZ* staining (blue) shows diffuse β-galactosidase transgene expression adjacent to metastatic B16-F10 melanoma cells (black cells, scale bar 30 µm). The white arrow indicates the area of micrometastases. (B) High magnification of the area in (A) indicated by the arrow showing diffuse *LacZ* staining in the area of metastases (scale bar 10 µm). (C) High magnification of a positive lymph node showing non-specific *LacZ* stain in an area devoid of melanoma cells. (D) Low magnification of a normal lymph node from an animal treated with NV1023. Only a non-specific *LacZ* staining is seen (scale bar 130 µm).

Histopathologic analysis was used to define positive (with melanoma metastases) and negative (no tumor cells) lymph nodes. The presence of NV1023 in sentinel lymph nodes with melanoma micrometastases was verified based on expression of the *lacZ* transgene. *LacZ* staining after NV1023 infection in vitro revealed cytoplasmatic expression of β-galactosidase (**Figure S1**). However melanoma metastatic cells in lymph nodes had dark pigmented cytoplasm, and it was difficult to see the light blue *lacZ* staining in the cells. Yet, by analyzing whole specimens of lymph nodes with metastases, we found diffuse accumulation of *lacZ* staining only in areas with metastatic melanoma cells (Fig 4A–C). In contrast, when the same staining was performed on normal lymph nodes of mice injected with NV1023, we noticed only light and non-specific staining of *lacZ* (Fig 4D). Similarly, positive lymph nodes treated with saline had no *lacZ* staining.

### [^18^F]FEAU PET imaging with NV1023 allows detection of sentinel lymph node metastases ***in vivo***

We randomly selected 5 of the tumor bearing animals for the test group. These mice were injected with 1×10^7^ PFU of NV1023 into the tumor-bearing and non tumor-bearing foot pads. In a second group of tumor-bearing mice (n = 5), the foot pads were injected with saline alone. This group served as a negative control. Two days after viral or saline injection, all mice received an intravenous injection of [^18^F]FEAU (250 µCi).

**Figure 5:**
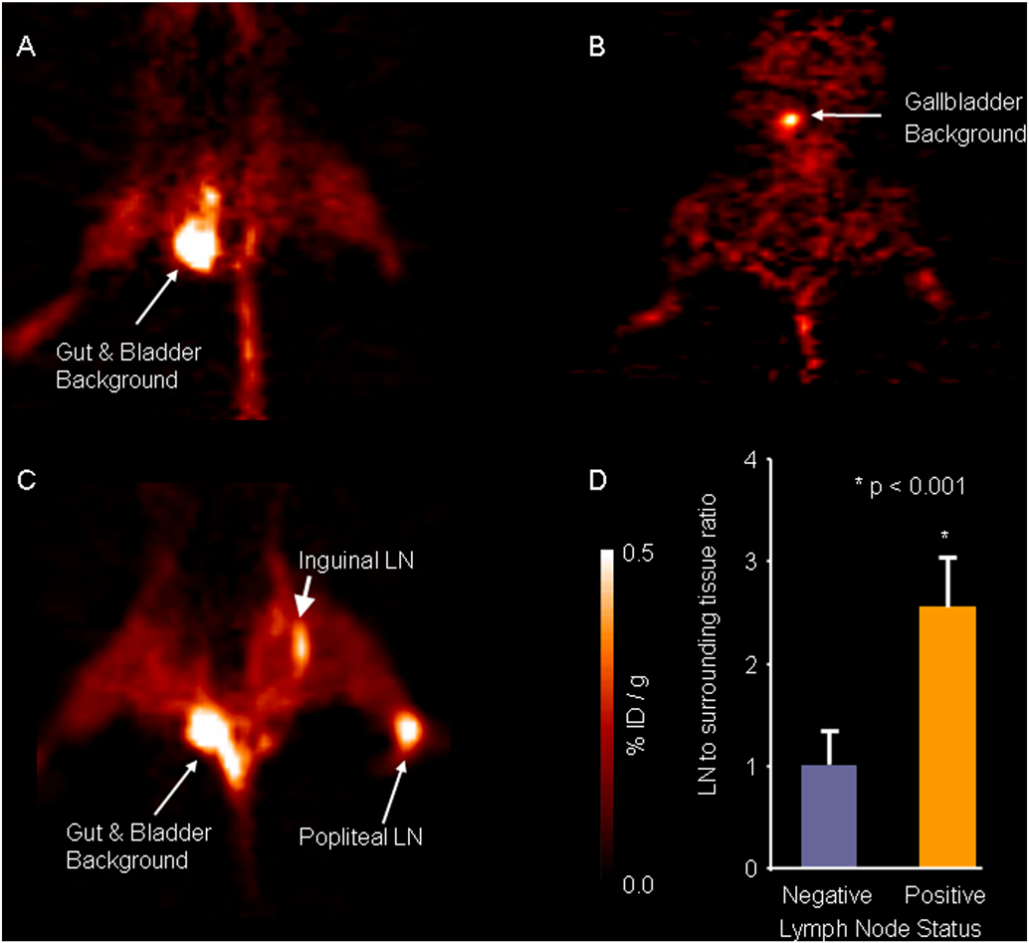
[^18^F]FEAU-PET imaging of mice with and without positive lymph node metastases. Representative coronal views of (A) a control animal with positive SLN injected with saline and (B) a mouse without tumor injected with NV1023, showing accumulation of radioactive signals in the gut, gallbladder and bladder. (C) A coronal view of a mouse with positive popliteal and inguinal lymph node metastases injected with NV1023. Positive PET signal was detected only in positive lymph nodes (white arrows). (D) The lymph node-to-surrounding tissue radioactivity ratios were measured in the PET scans; negative lymph nodes (blue column, n = 10), positive lymph nodes (orange column, n = 8).

Region of interest (ROI) measurements from [^18^F]FEAU PET scans in mice injected with NV1023 identified all tumor-positive lymph nodes (with melanoma metastases) (n = 8). Conversely, all tumor-negative lymph nodes in mice injected with NV1023 (n = 5) and mice treated with saline (n = 5) had [^18^F]FEAU signal levels similar to background (**Figure 5**). The lymph node-to-surrounding tissue radioactivity ratio was significantly higher (*P*<0.001) for tumor-positive lymph nodes compared to tumor-negative nodes. Nucleoside analogs are not accumulated or trapped by mammalian tissue, as only virus-containing cells can induce phosphorylation of these tracers by the HSV1-tk reporter gene [8], [15], [16], [17]. **Figure 5** shows a representative experiment of [^18^F]FEAU uptake in an animal with a lymph node metastasis injected with saline. The figure clearly demonstrates no radiotracer accumulation by mammalian tissue, aside from gallbladder uptake, where colonies of thymidine kinase forming bacteria may augment the background signal.

**Figure 6:**
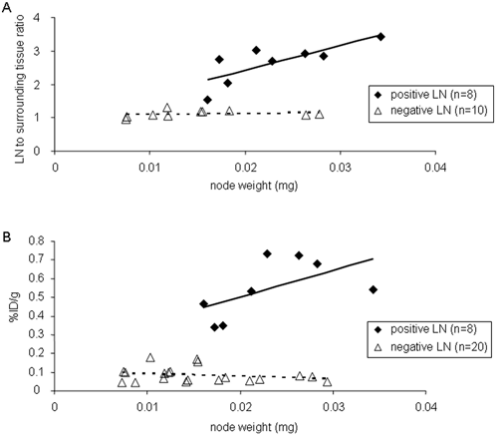
Radioactivity measurements and lymph node weight. (A) Lymph node-to-surrounding tissue radioactivity ratios obtained from the [^18^F]FEAU PET images in NV1023-treated animals *versus* lymph node weight (8 positive nodes (♦) R2 = 0.603; y =  74.0x+0.939 and 10 negative nodes (▵) R2 = 0.035; y = 2.62x+1.080). (B) *Ex vivo* radioactivity measurements from excised lymph nodes. Lymph node radioactivity (%ID/g) in NV1023-treated animals *versus* lymph node weight (8 positive nodes (♦) R2 = 0.328; y = 14.4x+0.214 and 20 negative nodes (▵) R2 = 0.054; y = −1.40x+0.108).

Immediately after imaging, the lymph nodes were excised, weighed and radioactivity analyzed in a gamma counter prior to histological examination. There was a clear separation in radioactivity levels (%ID/g) between tumor-positive (n = 8) and tumor-negative (n = 20) nodes with no overlap (**Figure 6B**). All positive lymph nodes had radioactivity values at least two standard deviations above the negative nodes. The radioactivity level profile is similar to that obtained from the ROI analysis of the microPET images (**Figure 6**).

Although statistical analysis revealed that the weight of tumor-positive nodes was significantly larger than tumor-negative nodes (0.023±0.006 and 0.015±0.006, respectively, *P*<0.05), the weight of 7 out of 8 positive nodes was in the range of the negative nodes (0.007–0.029 mg). This shows that NV1023-PET imaging system can identify tumor-positive lymph nodes within the range of ‘normal’ size lymph nodes, which is not the case with conventional imaging methods.

## Discussion

The standard of care for patients with intermediate and advanced stage melanoma is resection of the primary tumor with excision of first echelon lymph nodes that receive lymphatic drainage from the tumor [18]. Lymphoscintigraphy and SLN biopsy are used for staging of melanoma as well as other carcinomas [19], [20]. Currently, the search for SLN is performed either by use of blue dye injection into tumors resulting in the coloring of the draining nodes, or by injection of technetium-99m-labeled nanocolloid and localization of nodes by combining preoperative lymphatic mapping and intraoperative gamma probe detection [21]. These modalities for SLN mapping require surgery and pathologic analysis of the excised lymph nodes. The presence of metastases in one or more lymph nodes usually requires further treatment, which may include a second surgery, radiotherapy, immunotherapy, chemotherapy, or combination treatment [22]. In other mucosal tumors such as gastrointestinal and genitourinary carcinomas, tumors are not easily accessible for injection and lymph nodes are difficult to biopsy. Since occult lymph node metastases are common and have a major impact on survival, patients would benefit significantly from a noninvasive method that would allow accurate detection of lymph nodes invaded by cancer [2].

The imaging modalities used in clinical practice today for nodal staging include ultrasound, contrast-enhanced computed tomography, contrast-enhanced magnetic resonance imaging (MRI), and [^18^F]FDG PET scanning. Ultrasound, computer tomography and magnetic resonance imaging relay mainly on lymph node size. None of these methods can reliably detect subcentimeter lymph nodes with micrometastases [23], [24], [25].

Historical studies have shown that wild-type HSV-1 possesses a natural tropism for infecting tumor tissues [26]. Recent studies by our group and others have observed that oncolytic HSV-1 demonstrates a strong selectivity for infecting cancer cells including malignant melanoma cells [11], [12], [27], [28], [29], [30]. Although these viruses were not specifically designed to target malignant tumors, we have repeatedly observed that they display a remarkable selectivity for infecting malignant cells over normal cells [31], [32], [33]. This was also described in phase I studies for treatment of patients with malignant melanoma [34], [35]. The selective infection of cancer cells with oncolytic HSV was recently evaluated in our laboratory in association with normal stromal cells. Expression of viral reporter genes in cancer cells but not in normal stroma was detected in vitro and in vivo up to 7 days after infection [28].

Reporter gene imaging with HSV1-tk has been used extensively in animal models of cancer. The expression of HSV1-tk can be imaged and monitored using specific radiolabeled substrates that are selectively phosphorylated by HSV1-tk and trapped within transfected cells. Three radiotracers that are widely used by many investigators for imaging of HSV1-tk gene expression are [^18^F]FEAU, [^124^I]-2′-fluoro-1-β-D-arabino-furanosyl-5-iodo-uracil ([^124^I]FIAU), and 9-(4-[18F]fluoro-3-hydroxymethylbutyl)guanine ([^18^F]FHBG) [14], [36], [37], [38], [39], [40]. HSV1-tk–expressing bacteria have recently been shown to localize in various tumors, including B16-F10 murine melanoma cells, and primary tumors were successfully imaged with [^124^I]FIAU or [^18^F]FEAU [41], [42]. In a previous paper [8], we have shown that at 48 hours after [^124^I]FIAU administration, saline-treated tumors demonstrated a mean [^124^I]FIAU accumulation of 0.0021±0.0002 percent dose/g tissue, whereas tumors treated with 1×10^7^ p.f.u. of NV1020 demonstrated a mean accumulation of 0.0047±0.0004 percent dose/g (*P*<0.0002, n = 9 in each group). Similarly, tumors treated with 5×10^7^ p.f.u. of NV1020 demonstrated a mean accumulation of 0.0070±0.0009 percent dose/g (*P*<0.0002, n = 9). The specificity and efficacy of [^18^F]FEAU imaging using NV1023/HSV1-tk reporter gene was recently demonstrated by our group for various other tumors [28].

Here we describe a novel method for detection of occult SLN metastases using an oncolytic HSV/NV1023 and reporter gene imaging. Using NV1023 as the delivery vector, we demonstrate that a single injection of this attenuated, replication-competent virus into the primary tumor will induce expression of HSV1-*tk* in cancer cells invading lymph nodes that can be selectively imaged with [^18^F]FEAU PET. This imaging system was sensitive and accurate enough to detect micrometastases in lymph nodes that measured <3 mm in diameter. In one tumor-positive lymph node, the lymph node-to-surrounding tissue radioactivity ratio was close to unit, 1.5. This low ratio was primarily due to the high level of background radioactivity that surrounded this lymph node. This illustrates the importance of imaging when background radioactivity is low [43]. This imaging paradigm is capable of distinguishing between normal nodes and those infiltrated by cancer as early as 48 hours after viral injection. Although the sensitivity of this imaging paradigm (minimal number of tumor cells per node that are detectable) was not determined in this study, a significantly higher uptake and retention of [^18^F]FEAU was detected in all nodes invaded by cancer compared to non invaded normal nodes (>2 standard deviations).

Lymph node size may be correlated to immune infiltrates and not only to metastatic volume. We demonstrated that although tumor invaded lymph nodes were slightly heavier than normal nodes, the majority of lymph node with metastases (7 out of 8) were within the weight range of normal nodes. This suggests that lymph node weight alone can not predict the presence tumor micrometastases, and that NV1023-PET imaging system can identify tumor-positive lymph nodes within the range of ‘normal’ size lymph nodes, which is not the case with conventional imaging methods.

Studies have implicated the ability of oncolytic viruses, including HSV-1, to be effectively transported into the lymphatic circulation [44], [45], [46], [47], [48], [49], [50]. Herpesviral particles are 125 nm in diameter, and theoretically they can travel through the lymphatic system in the same fashion that malignant cells metastasize to regional lymph nodes. Injection of NV1023 viruses into normal skin was shown to result in viral transit to normal cervical lymph nodes [50]. In the absence of metastatic disease, NV1023 infection of normal cells within the lymph nodes was sparse and demonstrated rapid clearance from the lymphatic system [50]. However, in the presence of metastatic cells, viruses could be detected for several days after injection [47], [50]. Interestingly, herpesvirus may also travel to the lymph nodes within leukocytes or Langerhans cells [51], permitting viral replication and subsequent infection of nodal tumor cells. In addition, it is theoretically possible that residual melanoma cells at the primary tumor site may have been locally infected with virus, dislodged, and subsequently passed to the sentinel lymph nodes. It is also possible that both tumor cells and lymphocytes are being transduced in vivo. Nevertheless, in all three putative mechanisms, the end result would be selective expression of viral genes in positive lymph nodes [49], [52], [53].

Gene expression imaging of occult lymph node metastases was recently described by Burton et al. for direct PET visualization of prostate cancer [54]. In this study they used a prostate-specific adenoviral vector (AdTSTA–sr39tk) in conjunction with its tracer 9-(4-[^18^F]fluoro-3-hydroxymethylbutyl) guanine ([^18^F]FHBG) [55]. Peritumoral administration of this vector enabled direct detection of reporter gene expression in metastatic lesions within the lymph nodes. Detection of lymphatic metastases was previously described using oncolytic viruses carrying eGFP [45], [52], [56] or *Renilla* luciferase reporter genes [56]. In another study PET [^18^F]FEAU with HSV-1 oncolytic viruses was shown to enhance the detection of nerves infiltrated by cancer cells using noninvasive imaging [28].

None of the NV1023-treated animals suffered from clinically apparent side effects attributable to viral administration. The parent virus from which NV1023 was derived, R7020, has a very favorable safety profile in *Aotus* owl monkeys, a primate exquisitely sensitive to herpes viral infections [57]. Even at a 10,000-fold higher dose than wild-type HSV-1, R7020 remained less toxic to *Aotus* monkeys compared with HSV-1. A similar virus, NV1020, was recently studied in a phase I trial for patients with hepatic colorectal metastases. Doses of ≤1.3×10^9^ PFU were administered by hepatic infusion pump without dose-limiting toxicity or significant adverse events attributable to the virus [58]. NV1020 has a highly favorable safety profile, a finding that has encouraged our investigation of related vectors such as NV1023 for clinical application.

One potential diagnostic application of this viral-imaging paradigm is the staging of disease and guiding future treatment. In melanoma, head and neck, and breast carcinomas, imaging the targets of replication-competent viruses could be performed following a single intratumoral injection. In mucosal tumors such as gastrointestinal and genitourinary carcinomas, NV1023 or similar vectors can be delivered through endoscopic injections to intraluminal primary tumors [59]. Imaging of HSV-1-TK expression could then be visualized using a conventional PET imaging system to identify lymph nodes which harbor malignant cells. The ability of this method to detect micrometastases within SLN is likely to be better than conventional imaging techniques that require assessment of lymph node volume and non-contrast filling regions in the lymph nodes [60].

The high background signal in our system required amputation of the primary tumor and degutting of the animals previous to imaging. This problem may question the feasibility of HSV1-TK/[^18^F]FEAU imaging tool for diagnostic staging. In several human PET studies using nucleoside analogs and in our laboratory it was found that intestinal background uptake does not play an important role in patients (Brader and Blasberg unpublished data, [61], [62]. In addition, the relatively large distance of locoregional lymph nodes at the head and neck, axillary or popliteal regions from the intestines could further improve the signal to noise ratio in a clinical setting. In order to further reduce the radioactive background signal at the intestines, we are currently exploring different strategies to increase fecal elimination of radiotracer in a preclinical setting.

### Conclusions

Our study shows that the NV1023 herpes virus expressing the HSV-1-*tk* gene can track to draining lymph nodes following direct intratumoral injection and infect metastatic melanoma cells in the SLNs. Nodal metastases can be then successfully identified by [^18^F]FEAU PET imaging. The findings are encouraging and translation to the clinic is feasible. If successfully implemented, this imaging system has the potential for improving patient care by enabling: (1) a more sensitive noninvasive method for the detection of lymph nodes invaded by cancer, (2) delineation of the extent and anatomic location of regional metastases (3), provide better pre-surgical assessment of disease extent in patients with suspected positive lymph nodes and (4) provide guidance for adjuvant radiotherapy, immunotherapy, or chemotherapy to patients with lymph node metastases.

## Supporting Information

Figure S1 LacZ staining 48 hours after NV1023 infection in vitro revealed cytoplasmatic expression of beta-galactosidase.
(0.42 MB TIF)
