# Supplementary figures and images for: Imaging of Lymph Node Micrometastases Using an Oncolytic Herpes Virus and [18F]FEAU PET
Source: PLoS One. 2009 Mar 10;4(3):e4789. doi: 10.1371/journal.pone.0004789 (PMC2651472; doi:10.1371/journal.pone.0004789)

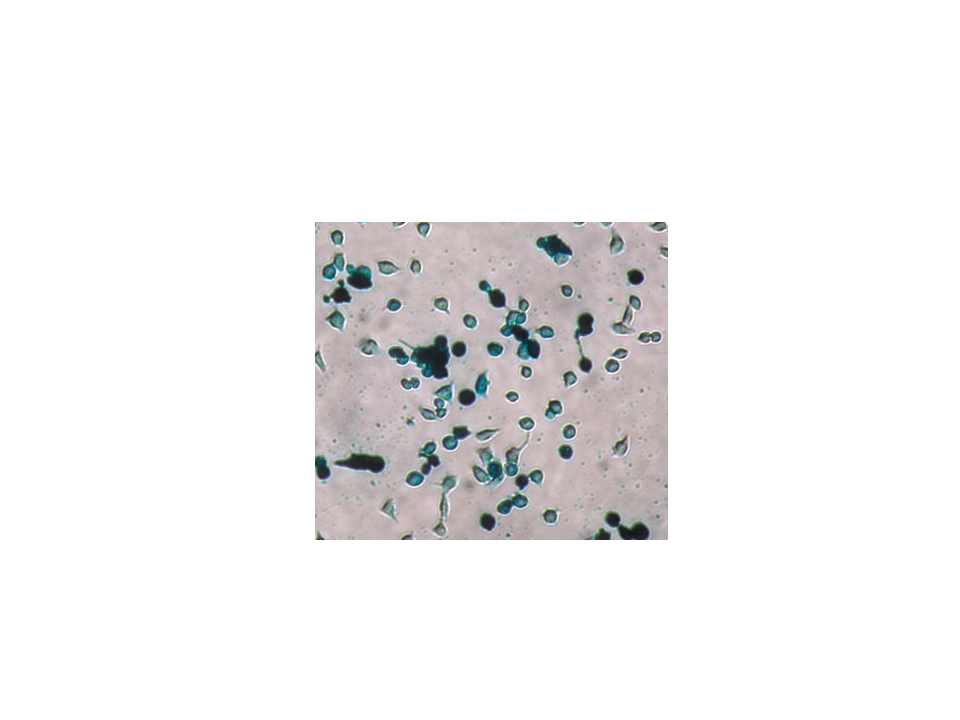

Supplement: Figure S1 — LacZ staining 48 hours after NV1023 infection in vitro revealed cytoplasmatic expression of beta-galactosidase. (0.42 MB TIF) [file pone.0004789.s001.tif]
